# Supplementary material for: Patterns of Intron Gain and Loss in Fungi
Source: PLoS Biol. 2004 Nov 30;2(12):e422. doi: 10.1371/journal.pbio.0020422 (PMC532390; doi:10.1371/journal.pbio.0020422)
Supplement: Table S1 — Also available at http://genes.mit.edu/NielsenEtAl/. (4.3 MB ZIP). [file pbio.0020422.st001.zip › NielsenEtAl/html/1240.html]

AN1094.1.NCU08980.1.MG06276.1.FG04130.1


```
 CLUSTAL W (1.82) Multiple Sequence Alignments - Introns Inserted


Sequence 1: MG06276.1	587 aa
Sequence 2: FG04130.1	575 aa
Sequence 3: NCU08980.1	577 aa
Sequence 4: AN1094.1	570 aa
Alignment Length: 590 aa
Number Identitical Residues: 311 aa
Alignment Score (without introns) 14730


MG06276.1 	MASARALARLAPQQARLSQVAQPAMRLQSQYRYMSMRTRRPVYSTPVTLQSQLQIQRRLE
NCU08980.1	MAST---SRALGRLS-APSMGVARLQTQAVSRLLSSAPRRALISESRQVAVTQQIRR---
FG04130.1 	MASS---ARAMTALSRVGHMAPSSRIAARALSTVSKTSARPSVAMRLNNTGRIAFRR---
AN1094.1  	MVGS------------TLAMRSNAMQLSSLSR-RSLTTRSPVQALRSSQSLSLRTARR--
          	*..:               :              *  .  .  :            *   

MG06276.1 	STEAPAATLSPQQPPKK---KFRKLRFLWRLIQLSLIGGLGYTAYSIYQERHPDEPADPD
NCU08980.1	----AHTETTPLPEPPKERRRFRKLRWLWRAPLFVSLAGIAYVGWGVYEERNPGPQVEPD
FG04130.1 	----AYADEAPKPRPGK---IRRTLRWTWRITYLSFGALVGYTCYVIYDDRHPQEQFQPD
AN1094.1  	----PYSDAAPLPAPKP-KKRFRFLRWTWRLTLLAGVGLAGNLAYNIYDQRHPTEQFVPD
          	    . :  :*   *       * **: **   :   .  .   : :*::*:*     **

MG06276.1 	PNRKTLVILG1TGWGSVSLMKNLDVENYNVIVISPRNYFLFTPLLPSCTTGTIEHRSIME
NCU08980.1	PSKKTLVVLG1TGWGSVSLLKKLDTEHYNVIVISPRNYFLFTPLLPSCTTGLIEHRSIME
FG04130.1 	PSKKTLVVLG1SGWGSVGLLKNLDTENYNVIVVSPRNYFLFTPLLPSCTTGLIEHRSIME
AN1094.1  	PSKKTLVILG1TGWGSVSLLKKLDTENYNVVVISPRNYFLFTPLLPSCTTGQVEHRSIME
          	*.:****:** :*****.*:*:**.*:***:*:****************** :*******

MG06276.1 	PVRTILRNKKAKVKYYEAEASSIDPDRKVVKIFDTSEVKGDMAETEVPYDMLVVGVGAEN
NCU08980.1	PIRTILRHKKANVKFYEAEASSVDPERKVVRVLDTSEIRGDVVETEIPYDMLVVGVGAEN
FG04130.1 	PVRTILRHKKGAVKYYEAEASSVDPDRKIIKIKDNTEGKGPQSETEIPYDMLVIGVGAEN
AN1094.1  	PIRSILRHKKAHVKFYEAEATKVDYEKRIVYISDDSEIKGDISHTEVPFDMLVMGVGAEN
          	*:*:***:**. **:*****:.:* ::::: : * :* :*   .**:*:****:******

MG06276.1 	ATFG~IPGVREHSCFLKEIGDAQLIRKKIMDCVETAAFKDQTPEEIDRLLSVVVVGGGPT
NCU08980.1	ATFG~IPGVREHTCFLKEIGDAQRIRKKIMDCVETAAFKGQSQEEIDRLLHMVVVGGGPT
FG04130.1 	ATFG~IPGVRENSCFLKEIGDAQLIRKKIMDCVERASFKGQSQEEIDRLMHMVVVGGGPT
AN1094.1  	ATFG1IKGVKENSCFLKEVGDAQRIRKRIMDCVETAMFKDQSEEEIKRLLHMVVVGGGPT
          	**** * **:*::*****:**** ***:****** * **.*: ***.**: :********

MG06276.1 	GVEFAGELADFFEEDIKKLIPEISDRFKVTLVEALPSVLPSFSKQLIEYTESTLKEEKIN
NCU08980.1	GVEFAGELQDFFEEDIKKLIPDIADRFRVTLIEALPNVLPSFSKQLIEYTESTFKEEKID
FG04130.1 	GVEFAGELRDFFEEDIKKLIPDISHRFKVTLIEALPNVLPSFSKQLIEYTENTMREENID
AN1094.1  	GVEFAGELQDFFEHDLRKWVPEIQENFRVTLVEALPNVLPMFSKQLIDYTESTFKEESIT
          	******** ****.*::* :*:* ..*:***:****.*** ******:***.*::**.* 

MG06276.1 	IETKTMVQKVTDKSVEATTTRPDGTKEKRVMPYGLLVWATGNAVRPLVKDLCARIPAQKD
NCU08980.1	IMTKTMVKRVTEKTVEAEISKPDGTREKITLPYGLLVWATGNAVRPVVKDLMERIPAQKD
FG04130.1 	IKLKTMVKKVTEDFVEAEFAGPDGSKQTLRIPYGLLVWATGNAVRPIVRDLMGKVPAQKD
AN1094.1  	IRTKTMVKNVTDKYIEAEVTKPDGTKELETIPYGLLVWATGNAIRPIVRDLMSQLPAQKN
          	*  ****:.**:. :**  : ***:::   :************:**:*:**  ::****:

MG06276.1 	SRRGLAVNEYLVVQGARDIWAIGDCAVAGYGPTAQVASQEGAFLARLFNNMAMTETLETK
NCU08980.1	SRRGLAVNEYLVVQGTRDIWAVGDCAVAGYAPTAQVASQEGNFLAGLFNNMARTEVLEQR
FG04130.1 	SRRGLAVNEYLVVQGTRDIWAVGDCAVAGYAPTAQVASQEGSFLARLFNNMAKTDTQEER
AN1094.1  	SRRGLAVNEYLVVNGTENVWAVGDCAITNYAPTAQVASQEGAFLARLFNTMAKTEAIEKE
          	*************:*:.::**:****::.*.********** *** ***.** *:. * .

MG06276.1 	IQELSSSLNVKQADRAPAEVSAEIAEYERKLRRIKDIRPFKYSHQGSLAYIGSDKAVADI
NCU08980.1	VRELSGSLNLAPGN--AAEISKEIEEHERQLRRIKDIKPFHYSHQGSLAYIGSEKAVADV
FG04130.1 	IKELSSSLNLKQGN--SAEIAAEIESLEKQLRRIKDVKPFRYSHQGSLAYIGSEKAVADV
AN1094.1  	LQRLSEAQSAAKSEEERNKIFDEIRDHQRQLRRTKQIGPFQYSHQGSLAYIGKERAVADI
          	::.** : .   .:    ::  ** . :::*** *:: **:***********.::****:

MG06276.1 	SWLDGNFATGGRLTYLFWRSAYLSMCFSA1RNRILVINDWVKSKIFGRDVSRE
NCU08980.1	SWFNGNLASGGSLTFLFWRSAYLSMCFST1RNRLLVINDWVKSKLFGRDVSRE
FG04130.1 	PWFNGNIASGGGLTYLFWRSAYLSMCFST1RNRVLVAVDWLKSKAFGRDVSRE
AN1094.1  	SWLSGNIASGGTMTYLFWRSAYLSMCFST1RNRVLVALDWVKAKLFGRDVSRE
          	.*:.**:*:** :*:*************: ***:**  **:*:* ********
```
